# A Streamlined In Vitro mRNA Production Evaluation for mRNA-Based Vaccines and Therapeutics

**DOI:** 10.3390/mps8060153

**Published:** 2025-12-18

**Authors:** Vittorio Madia, Sergio Minesso, Valentina Franceschi, Gaetano Donofrio

**Affiliations:** Department of Veterinary Science, University of Parma, 43126 Parma, Italy; vittorio.madia@unipr.it (V.M.); sergio.minesso@unipr.it (S.M.); valentina.franceschi@unipr.it (V.F.)

**Keywords:** Luciferin, IRES, encephalomyocarditis virus, cap analog, mRNA

## Abstract

To develop an effective strategy for in vitro mRNA production, it is crucial to evaluate the efficiency of the in vitro transcription platform. This can be accomplished using reporter genes, such as the luciferase-encoding gene. Luciferase activity assays provide a reliable means to assess the translation efficiency of in vitro transcribed mRNAs and to explore molecular dynamics associated with untranslated regions, capping, nucleotide analog incorporation, polyadenylation, and codon usage optimization. In this study, we propose a novel approach to performing the luciferase assay, offering a simpler, faster, and high-throughput method for evaluating in vitro generated transcripts to be employed for veterinary and human vaccine purposes as well as mRNA therapeutics

## 1. Introduction

The application of mRNA technology in COVID-19 vaccines played a key role in accelerating the development of mRNA-based gene therapies. Recombinant mRNAs can be designed and synthesized in vitro to exert biological activity in vivo. In vitro transcribed (IVT) mRNA is a single-stranded RNA molecule engineered to replicate the structure and function of endogenous mRNA [1]. Owing to its technological advantages, including a favorable safety profile, high efficacy, tunable immunogenicity, and ease of production and storage [2], IVT mRNA is increasingly recognized as a promising vector platform for therapeutic applications [3]. In order to obtain a similar resource, it is necessary to develop a platform that allows for efficient production of messenger RNA, first testing it on the expression of reporter genes.

Reporter genes are widely used in cell biology to investigate gene expression and associated regulatory mechanisms [4]. Among them, the gene-encoding luciferase, and its corresponding enzymatic assay, has emerged as a powerful tool for monitoring the functional dynamics of various genetic elements [5]. In particular, the *LUC2* gene, which encodes a human codon-optimized version of the firefly’s luciferase enzyme, can be placed under the control of a specific 5′ untranslated region (5′ UTR). This setup allows for the quantitative assessment of the 5′ UTR activity under defined experimental conditions [6]. The luciferase activity assay is, therefore, a highly effective method for evaluating the translational efficiency of messenger RNA synthesized in vitro. The standard workflow typically involves transfecting cells with mRNA, followed by an incubation period of approximately 24 h. Then, cells are lysed via detergent-containing buffer and luminescence is measured using a Luciferase Activity Reagent (LAR) applied to the cell lysate. Upon closer examination of the standard procedure, several limitations become apparent:

Sample preparation for luminometric reading is time-consuming.

The measurement is performed on lysed cells.

The protocol is not well-suited for high-throughput applications due to practical constraints in handling large numbers of samples.

The cost of reagents is relatively high.

Considering these challenges, a new protocol was developed to be simpler, faster, and more cost-effective. This alternative approach includes the following features:

Seeding cells in a white 96-well plate with a transparent bottom.

Using Luciferin as the substrate for luciferase instead of the conventional Luciferase Activity Reagent (LAR).

Measuring luminescence directly from live and intact cells rather than from lysates and maintaining cell viability is crucial, as it enables subsequent analyses such as omics approaches (including transcriptomics, metabolomics, genomics, epigenomics, etc.).

Reduced time and reagent costs for each measurement.

The method enables a high number of replicates per sample and allows for the simultaneous analysis of multiple samples, making it compatible with high-throughput screening.

The present method was used to compare the translational efficiency of various types of in vitro transcribed capped and uncapped mRNAs, since capping procedure is a limiting step during in vitro transcription.

## 2. Materials and Methods

### 2.1. Luciferin Method

The method described in this study involves seeding HEK293T cells (50 µL per well) in a 96-well plate with opaque (white or black) walls and a transparent bottom. Once cells were up to 70% of confluence, the culture media were removed and replaced with a transfection mix containing mRNA. Then, cells were incubated at 37 °C, 5% CO_2_ for 6 h. At the end of the incubation time, cells were supplemented with EMEM 20% FBS, then incubated at 37 °C, 5% CO_2_ for 24 h. For luminometric measurement, 50 µL of 10% Luciferin (D-Luciferin, Perkin Elmer (Waltham, MA, USA), 15 mg/mL in saline) diluted in EMEM was added in each well. Before measuring the light signals with a luminometer (Victor luminometer, Perkin Elmer), the plate was incubated in the dark for 3–5 min at room temperature to allow Luciferin to permeabilize the cell membranes. Data analysis and visualization were performed using GraphPad Prism (version 8.0.1, GraphPad Software Inc., 244 Boston, MA, USA).

### 2.2. Classic Protocol

HEK293T cells were seeded in a 24-well plate. Once cells were up to 70% of confluence, the culture media were removed and replaced with the transfection mix containing mRNA. Then, cells were incubated at 37 °C, 5% CO_2_ for 6 h. At the end of the incubation time, the transfection media were removed and replaced with fresh EMEM with 10% FBS, then incubated at 37 °C, 5% CO_2_ for 24 h. For luminometric measurement, the culture media were removed and cells were washed with PBS (Phosphate-Buffered saline, pH 7.4). 100 µL of Passive Lysis Buffer 1x (Passive Lysis Buffer 5X, Promega (Madison, WI, USA), diluted in water) was added in each well and then cells were incubated at −80 °C for at least 10 min. After cold incubation, cells were placed under agitation for at least 20 min and the lysates were then collected and centrifuged at 3000 rpm for 3 min. 50 µL of surnatant from each sample was placed in a black plate with 50 µL of LAR II reagent (Luciferase Assay Reagent II by Promega) for measurement with light luminometer (Victor luminometer, Perkin Elmer).

### 2.3. Cell Lines

HEKs (human embryo kidney cells) 293 T (ATCC: CRL-11268) were grown in complete Eagle’s minimal essential medium (cEMEM: 1 mM of sodium pyruvate, 2 mM of L-glutamine, 100 IU/mL of penicillin, 100 μg/mL of streptomycin, and 0.25 μg/mL of amphotericin B), supplemented with 10% FBS, and incubated at 37 °C/5% CO_2_ in a humidified incubator.

### 2.4. In Vitro Transcription

HighYield T7 RNA Synthesis Kit by Jena Biosence (Jena, Germany) was used for in vitro transcription following manufacturer protocol. To obtain capped transcripts, CAP5011 GAG (ENE) by Areterna Company (Bethesda, MD, USA) was used for co-transcriptional capping at a final concentration of 7.5 mM.

### 2.5. LiCl mRNA Precipitation

In vitro transcripts were precipitated and purified by 8M LiCl (Lithium Chloride) precipitation. To assess mRNA integrity, a bleach agarose gel was performed by adding sodium hypoclorite (0.12% final concentration) in agarose and TAE (Tris-acetate-EDTA) 1x Buffer [7].

### 2.6. Transient Transfection

An Easy Cell Transfection Kit by Synthgene Biotechnology Co. (Nanjing, China) was used for the transient transfection of in vitro transcribed mRNA on HEK293T cells. For the 24-well plate, 1 µg of RNA per well was used, whereas for the 96-well plate, 200 ng per well was employed following manufacturer protocol.

### 2.7. PCR and PCR-Mediated Mutagenesis

The 1250 bp ECD (extracellular domain) of rat Her-2 protein (RRT) was amplified from pCMV-RRTgD [8] with RRTgD106 sense (5′-[PHO] GGGCCACCATGATCATCATGGAGCTGGCGGCCTGG-3′) and RRTgD106 anti (5′- [PHO]GGGATCCTTAGGGGGAACCCCCATCGGCG-3′). The T7-IRESmut-ΔLUC2, obtained from pT7-IRES-LUC2 digestion with SmaI, was amplified with ATG-Mut-sense (5′-[PHO]GGGCCA**A**ATTATCAACGTGTTTTTCAAAGG-3′) and ATG-Mut-anti (5′-[PHO]GGGCTCGAGCTGGTACTGCATGC-3′). The PCR amplification reactions were implemented in a final volume of 50 μL, containing 20 mM Tris–hydrochloride pH 8.8, 2 mM MgSO_4_, 10 mM KCl, 10 mM (NH_4_)_2_SO_4_, 0.1 mg/mL BSA, 0.1% (*v*/*v*) Triton X-100, 5% dimethyl sulfoxide (DMSO), 0.2 mM deoxynucleotide triphosphate, and 0.25 μM of each primer. 1U of Pfu recombinant DNA polymerase (Thermo Fisher Scientific, Waltham, MA, USA) was used to amplify 100 ng of template DNA over 35 repeated cycles, including 1 min of denaturation at 94 °C, 1 min of annealing at 60 °C, and elongation at 72 °C (1 min and 30 sec for RRTgD106 and 3 min for pT7-IRESmut-ΔLUC2).

### 2.8. Plasmid Generation

pT7-IRES-LUC2 was generated and purchased from VectorBuilder Company (Chicago, IL, USA).

pT7-IRESmut-LUC2 was generated from reinserting LUC2, previously obtained from pT7-IRES-LUC2 digested with SmaI, in pT7-IRESmut-ΔLUC2 obtained from PCR amplification.

pT7-IRES-RRTgD was generated from inserting RRTgD106 amplified fragment (1250 bp) [8] in pT7-IRES-LUC2 digested with SmaI.

### 2.9. Western Immunoblotting

Western immunoblotting analysis was performed on protein cell extracts from a 6-well plate of HEK 293 T cells transfected with pT7-IRES-RRTgD, capped and uncapped, in vitro transcribed mRNA. For protein extraction, 100 μL of cell extraction buffer (50 mM Tris–HCl, 150 mM NaCl, and 1% NP-40; pH 8) was added to each pellet, and total protein quantification was performed using the BCA Protein Assay Kit (Pierce™, Thermo Fisher Scientific) following the protocol suggested by the manufacturers. The same amount of protein samples was electrophoresed on 10% SDS-PAGE and then transferred to PVDF membranes (Millipore, Merck, Darmstadt, Germany) by electroblotting. The membrane was blocked in 5% skimmed milk (BD), incubated for 1 h with primary mouse monoclonal antibody anti-glycoprotein D (clone 1B8-F11; VRMD, Inc., Pullman, WA, USA), diluted 1:10,000, and then probed with horseradish peroxidase-labeled anti-mouse immunoglobulin (A9044; Sigma, St. Louis, MO, USA), diluted 1:15,000, and finally visualized by enhanced chemiluminescence (Clarity Max Western ECL substrate, Bio-Rad, Hercules, CA, USA).

### 2.10. Data Analysis

The data were obtained from different mRNA transcription batches. For the conventional LAR system, which requires a 24-well plate and cannot be scaled down to a 96-well format due to the lysis and transfer steps, four distinct transcription batches were tested, each repeated once. Statistical analyses were conducted using Student’s *t* test and one-way ANOVA.

## 3. Results

### 3.1. “Classic” Method vs. “Luciferin” Method

As a first step, in order to validate the method, “Classical” and “Luciferin” methods were employed as models to rapidly assess the translational efficiency of two types of in vitro transcribed RNAs obtained from the pT7-IRES-LUC2 template, encoding luciferase in HEK293T cells. These transcripts differ in their 5′ untranslated regions (5′ UTR): one set carries only the EMCV IRES, known to be able direct translation in a cap-independent manner [9], while the other includes a trinucleotide cap analog plus the IRES.

**Figure 1:**
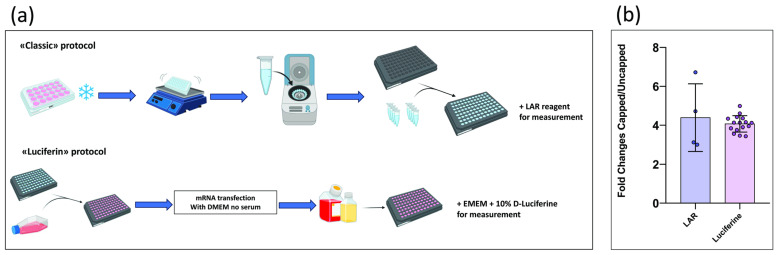
(**a**) Graphical comparison of the two protocols. Created with BioRender.com. (**b**) Comparison of the signals obtained from the two protocols. Signal intensity was assessed by calculating the ratio of luminescence values emitted by cells transfected with capped mRNA containing an IRES element (capped) versus uncapped mRNA containing an IRES element (uncapped).

Following the preliminary steps of cell seeding and subsequent transfection, the **classic protocol** (Figure 1a) involves the removal of the culture medium and the addition of 1x Passive Lysis Buffer. The plate is then incubated at −80 °C for at least 20 min, followed by agitation for an additional 20 min. The lysate from each well is collected and transferred to its corresponding microcentrifuge tube (Eppendorf, Hamburg, Germany). These are then centrifuged to pellet cellular debris and isolate the supernatant, which contains luciferase enzymes. The resulting lysate is dispensed into a black 96-well plate, and upon addition of LAR luminescence, it is measured using the Victor luminometer. In **the Luciferin protocol** (Figure 1a), cell culture, transfection, and luminescence measurement are, instead, all performed in the same plate. This is possible because a 96-well plate with opaque (white or black) walls and a transparent bottom is used. After transfection, cells are supplemented with MEM containing 20% FBS and incubated at 37 °C, 5% CO_2_ for 12–24 h. For measurement, MEM supplemented with 10% D-Luciferin is added to each well, the plate is incubated in the dark for 2–3 min, and luminescence signals are then recorded using the Victor luminometer.

The resulting luminescence data were used to perform a comparative analysis between the two approaches (Figure 1b), showing that the fold change between capped and uncapped samples remains consistent across both methods. Given this equivalence in experimental outcomes, all subsequent luminescence measurements were carried out using the **Luciferin protocol**, which offers increased practicality without compromising data reliability.

### 3.2. Capped mRNA vs. Uncapped mRNA

**Figure 2:**
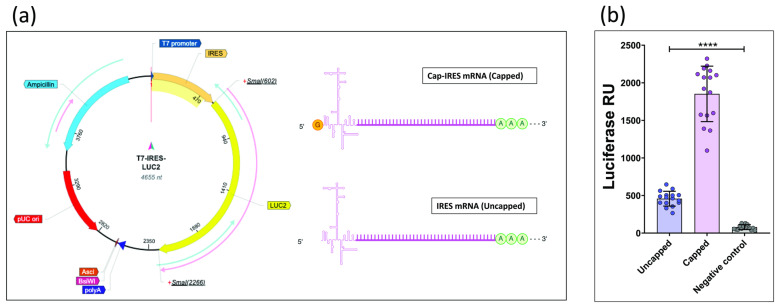
(**a**) T7-IRES-LUC2 and the two distinct transcriptional products. Created with BioRender.com. (**b**) Signals emitted by Cap-IRES mRNA HEK293T cells (capped) and by IRES mRNA HEK293T cell (uncapped) (****: *p* < 0.0001; one-way ANOVA).

Two distinct transcriptional products were generated from the same linearized DNA template (T7-IRES-LUC2) through in vitro transcription (IVT). The term “Cap-IRES mRNA” denotes transcripts incorporating a cap analog and the EMCV IRES sequence within the 5′ UTR, whereas “IRES mRNA” refers to transcripts containing only the IRES sequence in the 5′ UTR (Figure 2a). Given that mRNA capping is critical for both transcript stability and translational efficiency, these two processes were not distinguished in the present study. The resulting transcripts were transfected into HEK293T cells, and luciferase activity was subsequently quantified. A marked increase in luciferase activity was observed for capped mRNA compared to its uncapped counterpart (Figure 2b).

### 3.3. PCR-Mediated Mutagenesis of EMCV IRES

**Figure 3:**
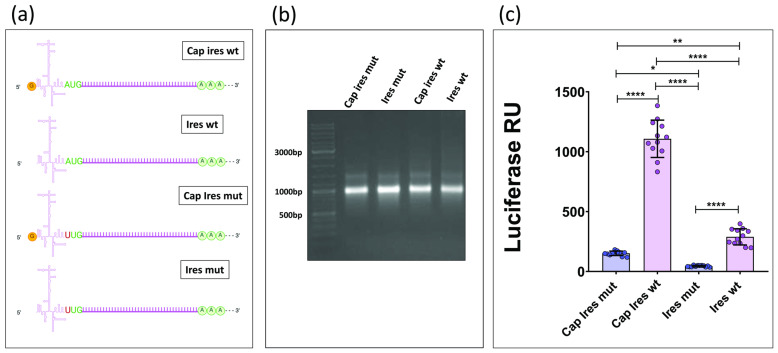
(**a**) The figure shows the four transcripts obtained and used for transfection. Created with BioRender.com. (**b**) Bleach agarose gel. (**c**) Luminescent signal emitted by HEK293T cells transfected with the four different mRNAs. (*: *p* < 0.02; **: *p* < 0.002; ****: *p* < 0.0001; one-way ANOVA and Tukey’s test).

The AUG11th site plays a pivotal role in EMCV IRES-mediated translation, serving as the direct ribosomal entry point without the involvement of a scanning mechanism [10]. To assess its impact on translation efficiency, this initiation site was silenced through PCR mutagenesis, leaving the LUC2 ORF ATG as the only available starting site for translation. This led to the generation of a new construct, T7-IRESmut-LUC2, from which two new types of in vitro transcripts (capped and uncapped) were produced, following the same protocol as before. Finally, four distinct transcriptional products were then generated (Figure 3a). The integrity of these four mRNAs was assessed via Bleach agarose gel (Figure 3b). Then, capped and uncapped transcripts containing the mutated IRES were compared to those with the wild-type IRES using a luciferase activity assay. (Figure 3c)

### 3.4. GTP Question: GTP 1.875 mM vs. GTP 7.5 mM

The evolution of cap analogs has led to the development of trinucleotide cap analog technology, which offers improved co-transcriptional capping efficiency and enables the generation of Cap 1 structures [11]. One specific issue we focused on is the need to dilute GTP concentration during the IVT reaction. This requirement arises from the competition between cap analog and GTP for the first position in the nascent transcript [12]. To address this, it is generally recommended to adjust the reaction so that the cap analog and GTP are present in a 4:1 ratio [13].

**Figure 4:**
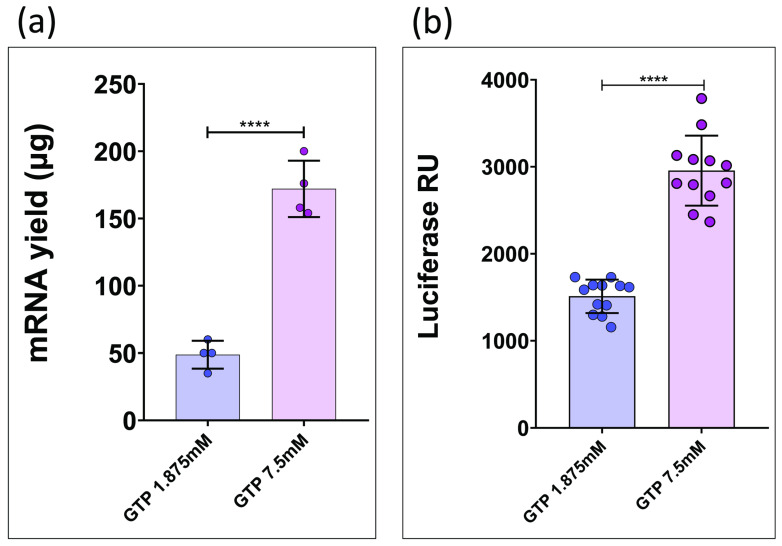
(**a**) Transcriptional yield data from two IVT reactions at different GTP concentrations, both performed using a trinucleotide cap analog. (****: *p* < 0.0001; *t*-student test). (**b**) Comparative luminescence data from HEK293T cells transfected with capped transcripts generated via IVT using 1.875 mM GTP versus 7.5 mM GTP. (****: *p* < 0.0001; Student’s *t* test).

However, this adjustment compromises overall reaction efficiency, resulting in lower RNA yields per IVT reaction. To further investigate this, we performed a series of IVT reactions using a trinucleotide cap analog and T7-IRES-LUC as DNA template. Some reactions were carried out with GTP at 1.875 mM (reflecting the 4:1 cap analog/GTP ratio), while others were conducted with GTP at 7.5 mM (Figure 4a).

The resulting transcripts were transfected into HEK293T cells and translation efficiency was assessed using a luciferase activity assay (Figure 4b).

### 3.5. Application of the mRNA Platform for RRTgD Expression

**Figure 5:**
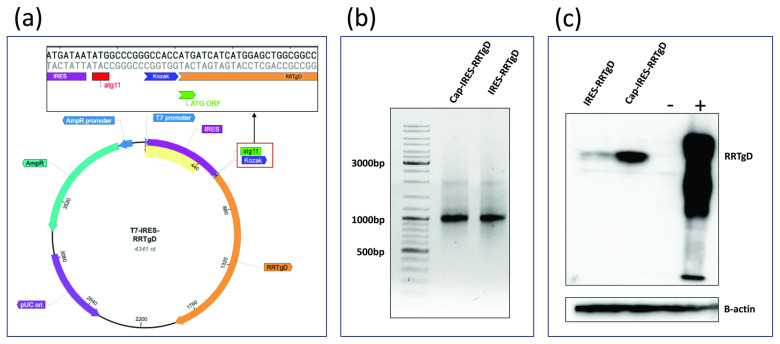
(**a**) Map of the T7-IRES-RRTgD construct, highlighting the in-frame alignment between the RRTgD106 ORF and the ATG11th of the EMCV IRES. (**b**) Bleach agarose gel. (**c**) Western blotting showing the expression of RRTgD106. β-actin serves as a loading control to confirm that the differences observed between samples are not due to loading errors. Western immunoblotting of extract from cells transfected with IRES-RRTgD and Cap-IRES-RRTgD mRNA. The lanes were loaded with 20 μg of protein extract. A negative control was established with HEK293T cells expressing GFP (−), whereas positive control (+) was established with HEK293T cells expressing an unrelated protein delivering the same tag of RRT-gD (gD106). Beta-actin was used as a loading normalizing control.

Luciferase and its derivatives are among the most reliable reporter systems for quantitative analysis in living cells. They are widely recognized as robust tools for evaluating transcription and translation processes, particularly for analyzing elements outside the open reading frame (ORF), such as untranslated regions (UTRs), capping structures, regulatory sequences, promoters, and polyadenylation signals. Insights gained from these preliminary assessments can then be applied to ORFs of specific interest. Therefore, the open reading frame (ORF) of LUC2 was replaced with the ORF of a tumor antigen tagged with an epitope from an animal herpesvirus (RRTgD106, obtained from pCMV RRTgD [8]), resulting in T7-IRES-RRTgD. Protein expression was evaluated by Western blotting. As shown in Figure 5a, the ATG start codon of the RRTgD ORF is in frame with ATG11th of the EMCV IRES sequence. The construct was then linearized and used as a template for IVT reaction, yielding a capped transcript and uncapped transcript. The integrity of the IVT products was confirmed by bleach gel electrophoresis (Figure 5b). Both transcripts were transfected into HEK293T cells, and protein expression was assessed via Western blotting (Figure 5c).

## 4. Discussion

Luciferase-based assays have long proven to be a reliable tool for investigating cellular and molecular dynamics [14]. However, their widespread application has raised a relevant question: *can this assay be further simplified in terms of laboratory practice*?

A brief review of the “classical” protocol reveals several critical steps that may impact the overall success of the experiment. One such step is the requirement to lyse cells and operate on the resulting lysate, a process that can become complicated when repeated measurements across multiple samples are required. Additional critical points emerge even in the early phases of the workflow, such as cell transfection and the subsequent replacement of the transfection medium with fresh medium. These steps, especially when working with cell lines highly sensitive to mechanical stress like HEK293T, can compromise the quality of the luminescence data collected. Considerations of time and cost also play a significant role in the feasibility of the classical approach. In contrast, **the “Luciferin” method** offers a simplified alternative. This protocol is performed entirely in a single 96-well black or white plate with a transparent bottom. Cell seeding, transfection, and luminescence measurement are all carried out in the same plate, offering several advantages, as described in the “Section 1” section:

The medium change step post-transfection is eliminated, thereby minimizing mechanical stress on the cell monolayer. Instead, the existing medium is simply supplemented with MEM + 20% FBS.

Measurements are conducted directly on living cells.

High-throughput analysis is facilitated by allowing multiple samples and replicates to be measured simultaneously in a single luminometric reading.

## 5. Conclusions

As shown in the Results section, the comparison between the classic and Luciferin methods gave equivalent experimental outcomes, allowing the use of Luciferin protocol as a simple and fast method for the analysis of in vitro transcribed mRNA. It was possible to confirm what is already known in the literature (such as the issue of GTP concentration in the reaction) and to gain further insights into the cap analog and EMCV IRES sequence dynamics for in vitro mRNA production in detail. When using trinucleotide cap analogs, maintaining GTP at a concentration of 7.5 mM is not only practical but also preferable. This is likely because trinucleotide analogs cannot be incorporated throughout the nascent RNA chain; instead, they can only be added as the first nucleotide during transcription initiation. From PCR mutagenesis results, the IRESmut sequence showed much lower translation efficiency compared to the wild-type IRES. These findings suggest that the ATG11th site is essential for EMCV IRES-mediated translation. Thus, as shown through both luciferase assays and Western blotting, EMCV IRES can support translation on its own in vitro, but not as efficiently as the cap analog. In order to achieve maximum translation efficiency in vivo, the use of the cap analog for in vitro transcribed mRNA is still essential.

## Data Availability

All data generated or analyzed during this study are included in this published article.

## Abbreviations

The following abbreviations are used in this manuscript:

IVT	in vitro transcription
UTR	Untranslated region
LAR	Luciferase Activity Reagent
HEK	Human embryo kidney
IRES	Internal ribosome entry site
EMCV	Encephalomyocarditis virus
ECD	Extracellular domain
RRT	Rat Her-2 protein
GTP	Guanosine Triphosphate
